# Hybrid Ceramic Membranes for the Removal of Pharmaceuticals from Aqueous Solutions

**DOI:** 10.3390/membranes11040280

**Published:** 2021-04-10

**Authors:** Jenny Radeva, Anke Gundula Roth, Christian Göbbert, Robert Niestroj-Pahl, Lars Dähne, Axel Wolfram, Jürgen Wiese

**Affiliations:** 1 Nanostone Water GmbH, Am Bahndamm 12, 38820 Halberstadt, Germany; anke-gundula.roth@nanostone.com (A.G.R.); christian.goebbert@nanostone.com (C.G.); 2 Surflay Nanotec GmbH, Max-Planck-Straße 3, 12489 Berlin, Germany; R.Niestroj-Pahl@surflay.com (R.N.-P.); l.daehne@surflay.com (L.D.); 3 Fachhochschule Magdeburg-Stendal, Institut für Wasserwirtschaft und Ökotechnologie, Breitscheidstr. 2, 39114 Magdeburg, Germany; axel.wolfram@h2.de (A.W.); juergen.wiese@h2.de (J.W.)

**Keywords:** water filtration, membrane technology, hybrid membrane, ceramic membrane, layer-by-layer coating

## Abstract

Layer-by-Layer (LbL) technology was used to coat alumina ceramic membranes with nanosized polyelectrolyte films. The polyelectrolyte chains form a network with nanopores on the ceramic surface and promote the rejection of small molecules such as pharmaceuticals, salts and industrial contaminants, which can otherwise not be eliminated using standard ultrafiltration methods. The properties and performance of newly developed hybrid membranes are in the focus of this investigation. The homogeneity of the applied coating layer was investigated by confocal fluorescence microscopy and scanning transmission electron microscopy (STEM). Properties such as permeability, bubble point, pore size distribution and Zeta potential were determined for both pristine and LbL coated membranes using various laboratory tests. Subsequently, a thorough comparison was drawn. The charging behavior at solid-liquid interface was characterized using streaming potential techniques. The retention potential was monitored by subjecting widely used pharmaceuticals such as diclofenac, ibuprofen and sulfamethoxazol. The results prove a successful elimination of pharmaceutical contaminants, up to 84% from drinking water, by applying a combination of polyelectrolyte multilayers and ceramic membranes.

## 1. Introduction

Providing consistently clean drinking water to the population is facing many challenges. The increasing global population and resulting worldwide rising pollution of surface water requires new approaches regarding sustainable water management. The supply of clean, pollutant-free drinking water is becoming increasingly difficult due to anthropogenic influences such as contamination with pharmaceutical residues (and catabolites, e.g., diclofenac, bezafibrate, carbamazepine, hormones, antibiotics, contrast agents) as well as specific contaminants such as agricultural pesticides [1]. The described problem is exacerbated by the increasing consumption of medication due to the increasingly aging societies of industrial countries. Besides, new problems resulting from increased use of micro- and nanoparticles and the stringent requirements for water quality (DOC (dissolved organic carbon) content) require new solutions for water treatment [2].

In the last decades, filtration as a material separation process became of great importance. Its benefits are low energy consumption, compact design, stable separation processes, modular setup with the possibility of a quick capacity expansion, rapid startup, and shutdown opportunities. Porous membranes made of plastics, ceramics, and metals are state of the art [2,3].

In the last decades, polymer membranes held the dominance for industry applications. They are inexpensive, easy to use and their pore size can be tuned to fulfill the application needs, up to the removal of all ions and trace elements from water as in reversed osmosis (RO) [4,5]. Disadvantages, however, are their low temperature resistance, their limited resistance to solvents and other chemicals for cleaning and therefore their short lifetime and low filtration performance per square meter.

Inorganic membranes, on the other hand, overcame the disadvantages of polymer membranes while at the same time also bearing good pressure stability, low fouling tendency, very good cleaning behavior, excellent possibilities for regeneration and high chemical stability [6]. These advantages enable a long service life with an extremely high operational reliability, long life expectancy and a high filtration performance per square meter [7,8].

Although membrane technology is widely used for water treatment, the separation of small molecules and ions is commonly not achieved by filtration processes. The necessary very small pore diameter for their removal leads to a significant decrease in the process flux and therefore of the process yield. Hence, methods such as ozonation, adsorption by activated carbon or UV-radiation are preferred for the separation of small molecules and ions [9,10,11]. The goal of this study is the combination of ceramic membrane technology with new innovative materials such as polyelectrolyte layers. The developed hybrid membranes may result in a product offering the possibility to filtrate the above-mentioned pollutants from wastewater without reducing the process yield.

Polyelectrolytes are polymers with dissociating groups in their chain. They can be divided into polycations, polyanions and zwitterionic polymers. The polyelectrolytes are used to improve the stability of aqueous colloids and gels, initiate flocculation and precipitation, and act as thickeners, dispersants, conditioners, emulsifiers, ion exchangers and clarifiers. They are used for the production of many shampoos, soaps, and cosmetics, as well as viands [12]. The properties of polyelectrolytes in solution are determined by the electrostatic interaction between the charged groups and the low molecular weight of ions in the solution [12].

The coating of polymeric membranes with polyelectrolyte multilayers (also Layer-by-Layer coating, LbL) has already been widely investigated [13,14,15,16]. To date, only a few commercial polymeric membranes with LbL technology are available, such as the dNF^®^ Membrane of NX Filtration BV and Membranes from X-Flow BV of Pentair.

On the other hand, the LbL-modification of ceramic membranes and its filtration efficiency on micropollutants are barely studied [17,18]. Due to their better performance in terms of long-term stability, chemical resistance, and low fouling tendency in comparison with polymeric membranes, the ceramic membranes may offer an expedient alternative for Layer-by-Layer (LbL) film modification.

LbL is a generally used process for coating substrates with functional thin films. The process flow of a multilayer structure production, whose driving force is the electrostatic interaction between oppositely charged polyelectrolytes [19,20], consists of four main steps representing a coating cycle [21]. By repeating the cycle [22,23], a desired number of layers can be applied to a given substrate. The entire thickness of the multilayer can therefore be controlled.

The most common cationic polyelectrolytes used in the LbL coating process are poly(allylamine hydrochloride) (PAH), poly(ethyleneimine) (PEI), and poly(diallyldimethylammonium chloride (PDADMAC)). The anionic polyelectrolytes used in LbL include mostly poly(vinylsulfate) (PVS), poly(acrylic acid) (PAA), and poly(styrenesulfonate) (PSS) [2].

The filtration performance of the coating is determined by parameters such as hydrophobicity, charge of the outer layer and density of the layer (the pore size). To use the effect of charge separation, the coating should have the same charge as the substance to be separated. Molecules which have the same charge as the membrane will be rejected because of electrostatic repulsion [24,25]. The size separation effect will remove substances having a larger diameter than the pores themselves. Hydrophobic molecules are easier to separate with a hydrophilic coating and vice versa.

The LbL coating has some basic requirements on the membrane material (substrate). The substrate must either be charged or should have functional groups for a covalent bonding of polyelectrolytes. A coating of uncharged, hydrophobic polymer membranes (e.g., PES) is also possible, but hydrophobic polyelectrolytes must be used, which can bind via hydrophobic interactions. Abtahi et al. [14] discovered the effect of hydrophobic substances binding to hydrophobic membranes until saturation.

The aim of this scientific work is to investigate the performance of ceramic-polymer hybrid membranes for a selected separation of hazardous substances from water, of which the elimination by using conventional membranes is currently not economically feasible. In the case of drinking water treatment, these are solutes of non-degradable compounds of natural origin as well as of anthropogenic origin. For that purpose, different polyelectrolytes were used on alumina membranes using the LbL strategy. Parameters such as ionic strength, pH values and molecular weight were varied, and their influence was characterized.

## 2. Materials and Methods

### 2.1. Raw Materials

**Figure 1:**
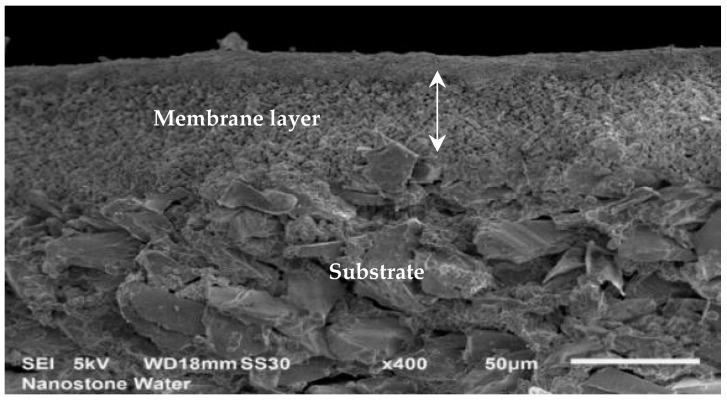
Cross-section profile of a ceramic mono-channel membrane.

Ultrafiltration ceramic membranes manufactured from aluminum oxide (Al_2_O_3_) by Nanostone Water GmbH were chosen for the substrate of the polyelectrolyte coat. The mono-channel tubes were selected due to their simple geometry, permitting an uncomplicated handling and coating process and a convenient approach for characterization. Aluminum oxide substrates were manufactured using a screw extruder of type D 120. The mono-channel tubes have an external diameter of 14 mm and an internal diameter of 8 mm. Their length is 300 mm. The filtration area corresponds to 0.075 m^2^ per 1000 mm. The ceramic membrane of the segments consists of double layer alumina particles (Figure 1). Before launching the LbL coating process, properties such as permeability, Zeta potential and filtration performance were characterized.

The LbL coating was performed using a combination of poly(allylamine hydrochloride) (PAH, MW 80–120 kDa, Sigma Aldrich, Darmstadt, Germany), poly(styrenesulfonate (PSS, MW 1000 kDa, Sigma Aldrich, Darmstadt, Germany) and Poly(diallyldimethylammonium chloride) (PDADMAC, MW 400–500,000 Da, Sigma Aldrich, Darmstadt, Germany). PAH is a water-soluble cationic polymer and finds applications in biomedical research and industry [26]. PSS finds usage as medication for high blood potassium treatment as well as anionic polymer in LbL coatings [27]. PDADMAC is mainly used in the wastewater treatment as coagulant and in the paper industry as controlling disturbing substances [28].

### 2.2. Polyelectrolyte Coating

The LbL coating was accomplished by repeating four main steps. Firstly, the purified negatively charged solid substrate is immersed in the solution of a cationic polyelectrolyte. Together with the adsorption, the electrostatic force forms the first polyelectrolyte layer of the multilayer structure. Subsequently, the excess and weakly adsorbed polyelectrolyte is removed from the surface by rinsing the substrate with deionized water. In a following step, the substrate is immersed in the solution of an anionic polyelectrolyte yielding after washing an approximately 2–6 nm thick bilayer structure. This step inverses the surface charge. By repeating this cycle, a desired number of layers can be assembled on the substrate. The entire thickness of the multilayer can therefore be well controlled [22,23].

**Figure 2:**
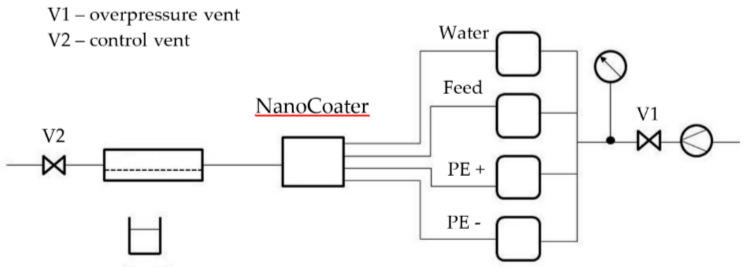
“NanoCoater”-system for coating the capillary membranes with polyelectrolytes.

LbL coating of the membranes was carried out using the NanoCoater of Surflay Nanotec, Berlin, Germany (Figure 2). The NanoCoater is an 8-channel automatic flow controller for liquids enabling the automatic coating with mixed polyelectrolyte compositions. The device is an optimized pneumatic system for coating pipes, hoses, membrane elements and microfluidics as well as for simply measuring the permeability and retention. Figure 2 shows the basic scheme for a simplified mono-channel pipe coating device. The polyelectrolytes were prepared as aqueous solutions and were transported through the membrane using air pressure. After each coating cycle, the membrane was rinsed with deionized water.

In detail, at first, four double layers of PSS_1000K_/PDADMAC_300–400K_ were coated in a solution with 0.5 M NaCl. This coating serves as a ground layer with high thickness and high permeability. Then, another four double layers of PSS_80–120K_/PAH_150K_ in a solution with 0.05 M NaCl and 10 mM tris(hydroxymethyl)aminomethane (TRIS) pH 7.0 are coated on the membrane. This layer serves as a filtration layer with smaller pores and higher hydrophilicity.

Between each coating step, the membrane was thoroughly rinsed with deionized water. The last step of production consisted of filtration with deionized water until the conductivity of the permeate was lower than 10 µS. Conductivity was measured with a Knick Portamess 911 Kond (Knick GmbH & Co. KG, Berlin, Germany) Conductometer. After this, the membranes were either directly tested or soaked in 15% glycerol for at least 4 h and then dried overnight. The conserved membranes are stable at room temperature until further testing.

### 2.3. Membrane Characterization

The developed hybrid membranes were subject to a different analysis in order to characterize their properties and behavior. Properties such as permeability, Zeta potential as well as filtration performance were analyzed for both LbL-coated and uncoated membrane and compared with each other. A measurement of the molecular weight cut-off with a combination of different polyethylene glycols by gel permeation chromatography (GPC) was performed after ensuring that no chemical reaction occurs between the cationic polymers and the chromatography column. Difficult for the analysis was the high water content of the polyelectrolytes. The samples should not be completely dried and diverse analyses such as measurement of contact angle were not possible.

#### 2.3.1. Surface Characterization

The surface of the hybrid membrane was firstly characterized using confocal laser scanning microscopy. To receive information about the distribution and penetration thickness of the polyelectrolytes, they were labeled with fluorescent dye rhodamine B (Rho). The fluorescence images were evaluated based on the fluorescence signal coming from the polyelectrolytes in the assembled LbL-film on the surface. Further analysis of the layer morphology was performed using scanning transmission electron microscopy (STEM) by Fraunhofer Institute for Microstructure of Materials and Systems (IMWS), Halle, Germany.

#### 2.3.2. Pore Size Distribution

Pore size distribution analysis was performed using permporometry for hybrid membranes and capillary flow porometry for the ceramic membranes. Capillary flow porometry can be used to characterize pore size from 13 nm to 500 μm and permporosimetry from 1 to 20 nm [19,20]. A non-reactive gas under increasing pressure is used to displace a wetting fluid from the sample in the capillary flow porometry. The equipment used for the analysis was Porometer 3 hz by Anton Paar GmbH, Ostfildern, Germany. The permporosimetry uses nitrogen which is wetted during the analysis until it reaches 90% saturation. The deviation of the specific flow as a function of the humidity of the applied gas flow characterizes the pore size distribution of the membrane. The analysis was performed by Fraunhofer Institute for Ceramic Technologies and Systems (IKTS), Hermsdorf, Germany.

#### 2.3.3. Molecular Weight Cut-off

Molecular weight cut-off (MWCO) is a widely used method for the characterization of membranes [29]. It is defined as the minimum molecular weight of a solute that is 90% retained by the membrane [29]. For the cut-off measurements of the LbL-coated membranes, technical polyethylene glycol mixtures (PEG) with various molecular weights (PEG 400–PEG 12,000 Da, each 1 g/L) were purchased at Merck, Darmstadt, Germany and used to determine a reliable cut-off. Dextran 500 kD (0.5 g/L) and Dextran 70 kD (0.2 g/L) mixture produced by AppliChem GmbH, Darmstadt, Germany was used for the uncoated membranes. The filtration for the feed and permeate sample was conducted in a laboratory filtration plant with a storage container of 7 l and an extern temperature control unit. The membranes were filtrated at 3 bar for 45 min at a cross-flow velocity of 3 m/s. The sample collection was carried out after the end of the filtration and was analyzed using gel permeation chromatography (GPC) with water as the eluent and calibrated with a PEG 100 standard. The analysis was performed by IKTS, Hermsdorf, Germany.

#### 2.3.4. Zeta Potential Characterization

Zeta potential gives important information about the forces which occur on a surface when it comes into contact with a fluid. It is defined as the potential which occurs on the slipping plane between any material and the ions from the liquid phase when they come into contact. The occurrence of an electrical potential is a result of shearing oppositely charged ions away from the electrochemical double layer by a streaming liquid medium [30].

The Zeta potential of the membranes was characterized through the surface streaming potential occurring during a flushing process of the surface with low concentrated solutions of electrolytes. By applying the Helmholtz–Smoluchowski equation, Zeta potential may be expressed as a function of the streaming potential U_str_

(1) $$\varsigma =({\mathrm{dU}}_{\mathrm{str}})/d\Delta p*\eta /(\varepsilon *\varepsilon_{o})*L/A*1/R,$$

where ε_0_ is the vacuum permeability, ε is the fluid dielectric constant, η is the viscosity of the fluid, L is the length of the rectangular slot channel between two flat surfaces, A is the cross-section area of the rectangular slot channel between two flat surfaces and R is the electrical resistance in the flow channel [22]. The device used for this analysis was SurPass Eco by Anton Paar GmbH, Graz, Austria.

The second method used in this work for characterization of the Zeta potential of suspension is electrophoretic light scattering. The filtration layer of a ceramic membrane (Nanostone Water GmbH, Halberstadt, Germany) was removed and ground into microparticles using a mortar and pestle. The microparticles were washed with water and centrifugated. The particles were then disperged with an ultrasonic cup booster (Bandelin Sonopuls, Berlin, Germany). After this, the particles were coated with PDADMAC and PSS with intermediate washing and centrifugation (3 times washing in ultrapure water). After each coating step, the charge of the particles was measured with a Zetasizer Nano ZSP (Malvern Panalytical, Malvern, UK) in 1 mM TRIS Buffer pH 7.

The Zeta potential of particles was calculated by Helmholtz–Smoluchowski equation as well.

#### 2.3.5. Filtration Experiments

Optimal filtration conditions should be aimed at maximum membrane permeability without losing selectivity. The membrane permeability can be diverted from the law of Hagen–Poiseuille for a porous structure [31]:

(2) $$L=(\epsilon_{p}*d_{\;p}^{2})/32\eta x,$$

where L is the membrane permeability, ϵ_p_ is the membrane porosity, d_p_ is the pore size, η is the dynamic viscosity of fluid, and x is the membrane thickness.

**Figure 3:**
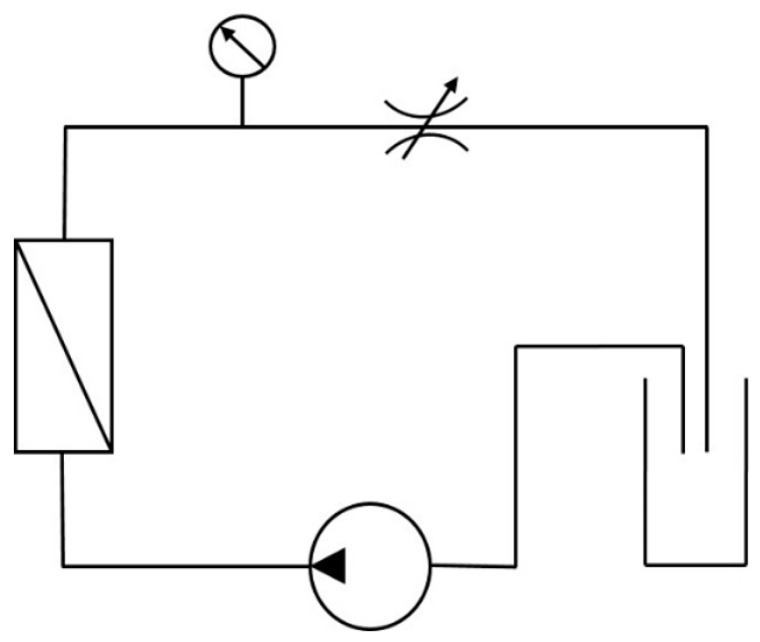
Scheme of the filtration system.

The experiments were done in a lab-scale filtration plant manufactured by IKTS (Hermsdorf, Germany). The plant had the size of 85 × 71 × 86 cm and is designed as a single pump filtration system for aqueous systems in a range between 0 and 30 bar and a high variety in stream velocities. The main corpus consists of stainless steel V4A but due to the lower thermal resistance of the used gaskets the temperature can be varied between 5 and 60 °C. The system can be operated in a closed cycle mode or as batch operation (Figure 3).

Crossflow velocity was 0.732 m/s, calculated from volumetric flow rate and the diameter of the mono-channel membranes. The corresponding Reynolds number is 1000, which means the flow pattern is in a laminar regime. The applied pressure was varied between 2 and 4 bar pressure.

Filtration experiments were conducted with a mixture of pharmaceuticals whose incidence in drinking water has been confirmed in multiple studies [9,10,11]. The tests were performed with a solution of ultrapure water with 600 mg/mL magnesium sulfate (MgSO_4_), ibuprofen (IBU) (M = 206 g/mol), clofibric acid (CLO) (M = 215 g/mol), sulfamethoxazole (SMX) (M = 253 g/mol) and diclofenac (DIC) (M = 296 g/mol) with a concentration of 0.1 mg/L. The rejection rates were monitored using high-performance liquid chromatography (HPLC).

Using Equation (3), the rejection by the membrane can be easily calculated from the ratio between the concentration of the feed and its concentration in the permeate for every tested substance.

(3) $$R\left(\%\right)\;=\;(1-C_{\mathrm{pi}}{/C}_{\mathrm{Fi}})\times 100\%$$

where R is the rejection, C_pi_ is the concentration of salt or contaminants in the permeate, and C_fi_ is the concentration of contaminant in the feed.

Permeability W was calculated according to the formula:

(4) Permeability (W) = V/(A * t * p) ,

where W is the membrane permeability (L·m^−2^·h^−1^·bar^−1^), V is the volume of the sample, A is the membrane area, t is the filtration time, and p is the pressure. Therefore, the normalized permeability can be found as the ratio between the permeability over filtration time t (Perm_t_) and the initial permeability of pure water (Perm_i_).

(5) $$\mathrm{Normalized}\;\mathrm{permeability}\;=\;\frac{{\mathrm{Perm}}_{t}}{{\mathrm{Perm}}_{i}}$$

The ionic strength of the solution was adjusted using MgSO_4_ in order to provide an ionic background strength, to optimize the filtration process and to achieve a better yield.

## 3. Results and Discussion

### 3.1. Surface Characterization

To evaluate the success of the coating process, random samples were subjected to fluorescence microscopy analysis and a combination of focused ion beam (FIB) and scanning electron microscope (TSEM).

**Figure 4:**
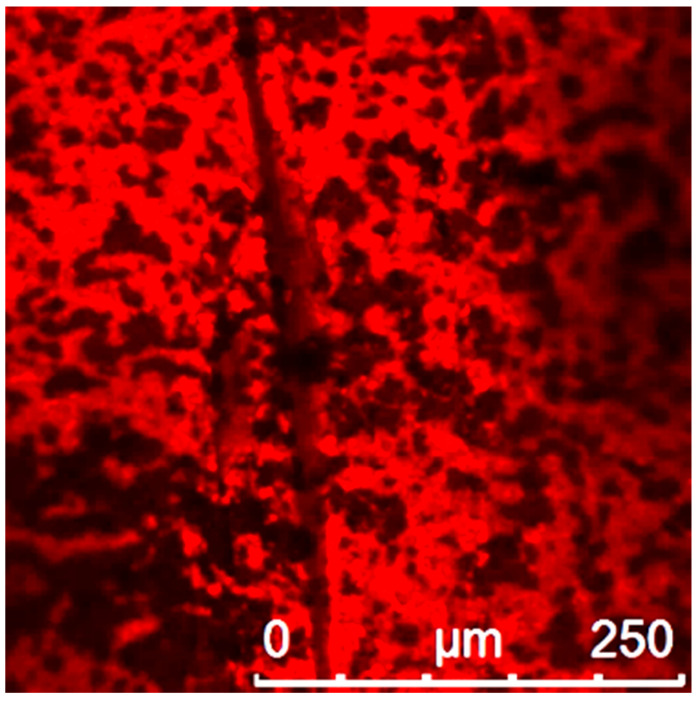
Confocal microscopy image of a hybrid membrane with the last layer of poly(styrenesulfonate) (PSS)-rhodamine 70 K. Voltage used for the photomultiplier tube (PMT)—700 V.

For fluorescence analysis, the polyelectrolyte layers were marked with an additional polyelectrolyte conjugated to small fluorescence molecules (Rhodamine). Based on the fluorescence intensity, the homogeneity of the polyelectrolytes was characterized (Figure 4).

Based on the result in Figure 4, one can conclude, that the polyelectrolytes were able to get attached to the membrane surface. The structure is porous but seems to have uncoated patches. This depends on the narrow depth of field of the confocal microscope. If the focus is shifted, the darker patches are also showing fluorescence. Overall, the surface of the membrane seems quite rough.

When an ionic compound is dissolved in a solvent, the ions shield charges on the polyelectrolyte chains. The ion concentration in the solution determines the film-forming properties of the polyelectrolyte as well as the conformation which the polymer has in solution. With increased ionic strength in the coating solution, more charges in the polyelectrolyte will be screened and the overall charge of the polyelectrolyte is reduced. This leads to the effect that in the same area more polyelectrolyte molecules will bind, to equilibrate the surface charge. Therefore, the fluorescence signal of the surface coated with polyelectrolytes strengthens.

Contrastingly, in a salt-free solution the polyelectrolyte molecules are almost not screened by ions and have a more linear structure because of charge repulsion in the molecule. With less or no salt, fewer polyelectrolyte molecules will bind to the surface because the molecules will form a less dense and more two-dimensional layer on the surface [32,33].

**Figure 5:**
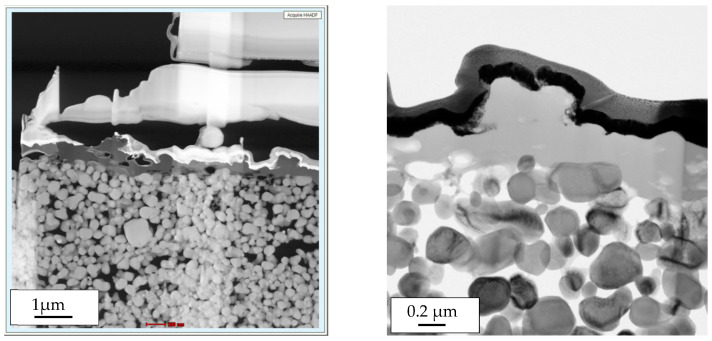
Scanning transmission electron microscopy (STEM) images of the Layer-by-Layer (LbL) coat of the ceramic support membrane.

The morphology and the thickness of the applied LbL layer was characterized using STEM and HAADF (high angle annular dark field) detector (Figure 5). The analysis confirmed that the LbL layer is morphologically homogeneous. The single polyelectrolyte layers are not visible and there are no further inhomogeneities included in the layer. No micro-cracks or delaminations were detected. The measured thickness of the layer varied over the surface and has an average value of 100 nm.

### 3.2. Pore Size Distribution

**Figure 6:**
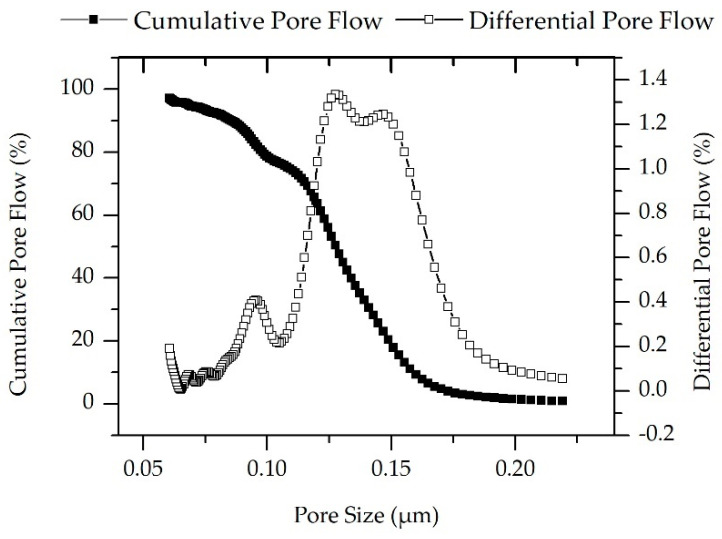
Pore size of the ceramic membrane as a function of the differential gas flow and cumulative gas flow through the pores, measured using capillary flow porometry.

After confirming successful coating, the pore network formed by the polyelectrolyte was analyzed by its mesh size. As a reference to the hybrid membrane, the pore size of the ceramic membranes was characterized first. The estimated mean pore size of the ceramic elements measured using capillary flow porometry was 128 nm, as shown in Figure 6. Due to the multilayer structure, it is not possible to precisely measure only the pores of the membrane (filtration) layer. The smallest pore size (expected in the membrane layer) and maximum pore size (expected in the support layer) were, respectively, 59 and 215 nm. The results are summarized in Table 1.

**Figure 7:**
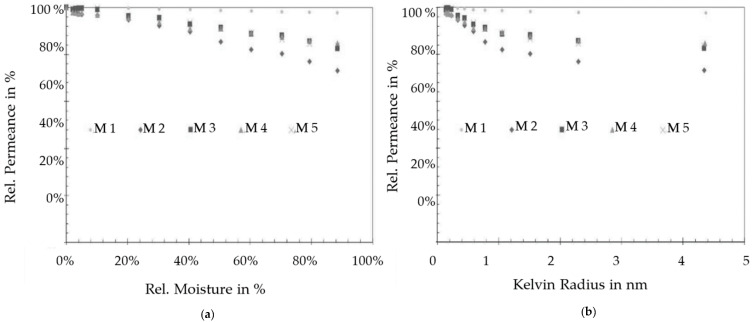
Relative permeance of a hybrid membrane as a function of (**a**) the relative moisture of the nitrogen gas flowing through the pores; (**b**) the calculated Kelvin radius of the pores.

The permporometry investigation of the hybrid membrane started with determination of the dry gas permeance at a pressure between 400 and 800 mbar. The detected values in five measurement cycles were between 0.3 and 7.8 kg/m^2^ hbar. In the next step, the nitrogen gas flowing through the membrane was gradually moistured with water, until all the pores were closed and no mass transport occurred. The permeance of the membranes was determined for each moisturizing rate. In Figure 7a are given the relative permeances as a function of the nitrogen stream humidity for five membranes (M1–M5). In Figure 7b, the relative humidity was converted into the Kelvin radius using the Kelvin equation.

The tested membranes showed pore blockage between 2.5% and 85% based on the moisture rate. One can conclude that 15% to 97.5% of the mass transport through the membranes occurs through pores larger than 4.4 nm. Due to the multilayer structure (ceramic support, double ceramic membrane layer and LbL coat on the top), it is not possible to measure only the mass transport of the LbL layer and therefore the results summarize the situation for the complete membrane system. As shown in Figure 7b, the pore blockage of all tested membranes has its maximum at values smaller than 1.5 nm Kelvin radius. According to these results, 10% to 20% of the mass transport in the membrane occurs through pores with Kelvin radii of less than 1.5 nm and approx. 84% or approx. 73% of the mass transport takes place through pores with Kelvin radii of greater than 4.4 nm.

The investigation of the pore size distribution confirmed that the polyelectrolytes were able to form a pore network over the ceramic membrane with Kelvin radii of about 1.5 nm, which causes the strong blockage in this range. Nevertheless, the results represent a plot of mass transport through the complete membrane and therefore further investigation of the LbL network is necessary.

### 3.3. Molecular Weight Cut-Off (MWCO)

The investigation of both membrane types—ceramic and hybrid—went further with the testing of their MWCO with commonly applied substances. The ceramic membranes have larger pores and therefore their retention was characterized using substances with a larger molecular weight—a homogeneous mixture of Dextran 500 (MW between 450,000 and 550,000 Da) and Dextran 70 (MW between 65,000 and 75,000). The prepared solution was streamed through the membrane at 3 bar pressure for 45 min. The permeate was collected at the end of the filtration cycle. After 45 min of filtration, the mixture of Dextran 500 and Dextran 70 started to precipitate. Therefore, no further chromatographic investigation of the permeate for ceramic membranes was possible. The reasons for this undesired reaction are to be further investigated. As an outlook, further substances with a well-defined molecular weight must be selected and used for MWCO characterization of the ceramic membranes.

PEG mixture was used for MWCO determination of the hybrid membranes. Like in the case of ceramic membranes, the mixture was filtrated for 45 min at 3 bar. Subsequently, the pressure was increased gradually up to 9 bar in order to promote mass transport through the membrane. The mixture of PEG was measured using gel permeation chromatography. Before testing the permeate with gel permeation chromatography, its conductivity was measured to ensure stability of the LbL system and lack of back-washed cationic polymers.

**Figure 8:**
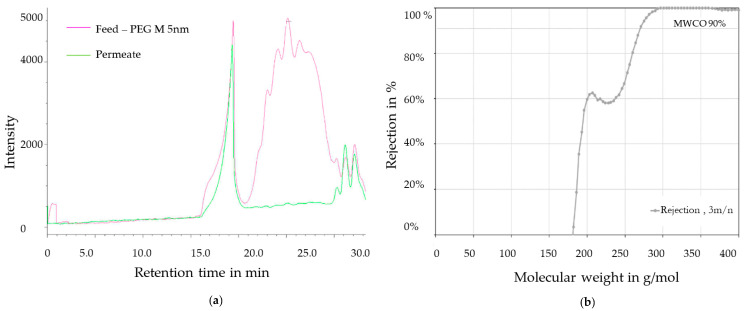
(**a**) Chromatogram of polyethylene glycol (PEG) mixture before and after filtration using a hybrid membrane. (**b**) Rejection of PEG as a function of their molecular weight.

Figure 8a shows the chromatogram of the sample before and after filtration. Before conducting the experiment, the PEG peak is clearly visible in the diagram. After the filtration cycles, the retention of the different PEGs was calculated as a function of the reduced intensity of the peaks of each PEG type. The calculated retentions are shown in Figure 8b. The chromatographic investigation of a permeate sample from the hybrid membrane, received during filtration at 3 to 9 bar with a calibrated mixture, showed 90% retention of PEG with a molecular weight of 270 g/mol (≈Da). The unsteady behavior in the range < 240 g/mol is due to the peak distribution in the chromatogram back from minute 30.6—it can be considered a process-related calculation misconception. The diagram range < 240 g/mol is therefore not representative.

According to the received results for pore size distribution and molecular weight cut-off, the hybrid membrane can be qualified as a nanofiltration membrane [26]. 

### 3.4. Zeta Potential

The physical size of the membrane pores is not the only factor promoting rejection. Active groups on the surface may reject or attract loaded molecules or ions from the liquid solution and in this way contribute to the rejection rate of a membrane. The electrostatic potential of the surface and its interaction depends strongly on the pH and ion concentration of the solution. The goal of this study was to develop a hybrid membrane with switchable electrostatic potential, which may be adjusted to the selected application. In order to characterize the membrane surface before and after coating, its streaming potential was measured in a series of experiments. The application of LbL coatings should lead to the formation of new active groups on the surface of the membrane and therefore to a change of the streaming potential of the membrane.

**Figure 9:**
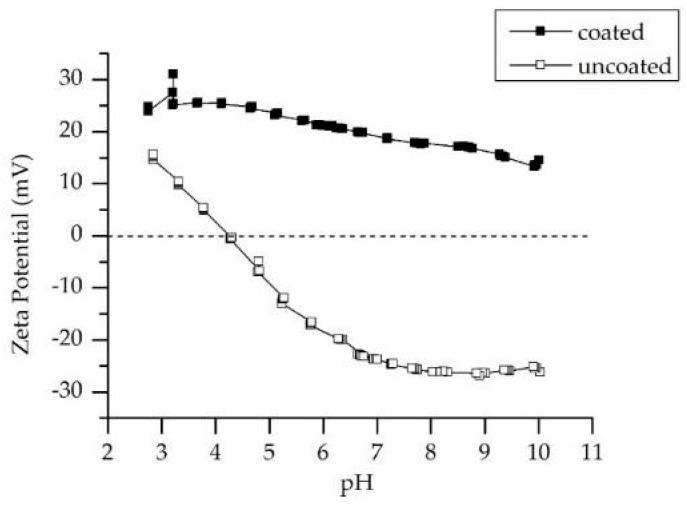
Zeta potential as a function of pH for an alumina ceramic membrane and LbL-coated alumina ceramic membrane.

Figure 9 shows the Zeta potential scan of an original Al_2_O_3_ ceramic membrane without an LbL coat in comparison to a pH scan of the hybrid membrane. The scan was performed from pH 10 to pH 3 by titrating the water solution with 0.01 M potassium chloride. The scan shows that the ceramic membrane is has negative Zeta potential in the largest part of the pH range. Its isoelectric point or the pH at which a molecule carries no net electrical charge or is electrically neutral in the statistical mean [30] is detected at a pH of 4.16. After applying the LbL coating on the ceramic surface, a pH scan was performed under the same conditions and the results were compared with the values for standard alumina membranes. In contrast to standard alumina, the coated membrane has positive Zeta potential values in the range between pH 3 and 10. No isoelectric point was detected in this pH range.

The modification of ceramic alumina membranes with polyelectrolyte multilayers leads to a change of the surface properties of the membrane and therefore influences the repulsion and attraction forces, which may occur between the surface and contaminants.

It must be noticed that the detected positive values for the hybrid membrane do not necessarily mean a positively charged surface. The polyelectrolytes attached to the surface are strongly charged and influence the ionic strength of the system. An increment of the ionic strength influences the Zeta potential and shifts its values into the positive range [34]. Therefore, the measured Zeta potential cannot be used for comparison with the filtration behavior of the membrane in later research. Furthermore, the used salt for the filtration experiments was MgSO_4_. MgSO_4_ has two valent ions, which reduced the sensitivity of the measuring technique [34], and therefore the measuring of the Zeta potential in the presence of MgSO_4_ cannot be representative.

**Figure 10:**
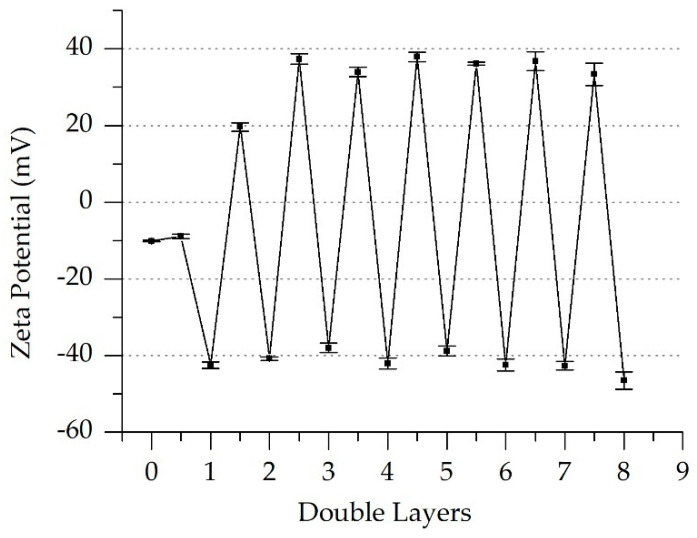
Zeta potential of a ceramic microparticle’s suspension in the presence of polyelectrolyte multilayers (PSS/PAH).

In comparison, the Zeta potential of the polyelectrolyte used for the last coating cycle was measured using the electrophoretic mobility in the presence of the suspension of ceramic partricles. As shown in Figure 10, the measured Zeta potential of the suspension showed a clear negative potential for the last layer (PSS, Double Layer 8). The bare ceramic parcticle suspension has a low negative charge (Layer 0). Beginning with layer 1.5, the polyelectrolytes flip the charge of the particles with each layer. Further investigations are needed to characterize the charging behavior of the hybrid membrane.

### 3.5. Permeability and Rejection

The permeability and rejection of the hybrid membranes were characterized in two series of experiments. At first, the permeability of the ceramic and hybrid membranes was determined and compared. In the second series of experiments, the rejection of water-based solutions of various pharmaceuticals was characterized.

**Figure 11:**
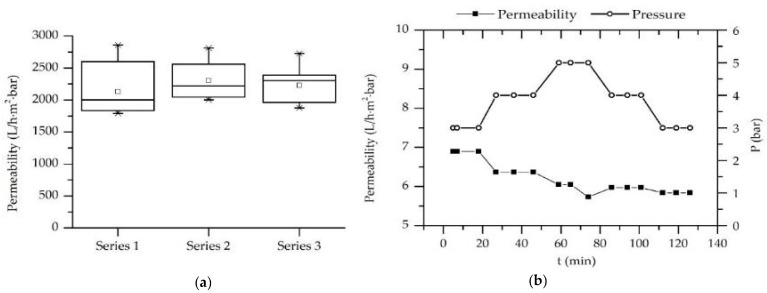
(**a**) Permeability of ceramic monotubes. (**b**) Permeability of a hybrid membrane as a function of pressure and filtration time.

Figure 11 shows the permeability of ceramic monotubes (filtration area of 0.075 m^2^) produced by Nanostone Water GmbH. The permeability was measured three times for every series to achieve more precise results. The test was performed at 2 bar pressure for 30 min. As shown in Figure 11a, the measured permeability is between 2130.83 and 2303.23 L/m^2^·bar·h. The standard deviations were in the range between 250 and 340 L/m^2^·bar·h. The large standard deviations are to be led back to the testing procedure—the permeate was collected directly from the filtration unit and measured in a graduated jug.

Figure 11b shows the permeability of the hybrid membrane as a function of the pressure and test duration. The applied pressure was manually varied over the time to monitor its influence on the permeability. The results revealed a strong decrease in the membrane permeability due to the LbL coating. Considering the strong reduction of the membrane pore size, the decrease in the permeability was an expected effect. No correlation between permeability and pressure was found. The slight decrease in the permeability with the test duration (from 7 L/m^2^·bar·h in the beginning of the process to 5.8 L/m^2^·bar·h after 120 min testing) is to be explained with the running-in characteristic of the membrane, which characterizes the processing time for the settling of a continuous mass transport regime through the membrane.

**Figure 12:**
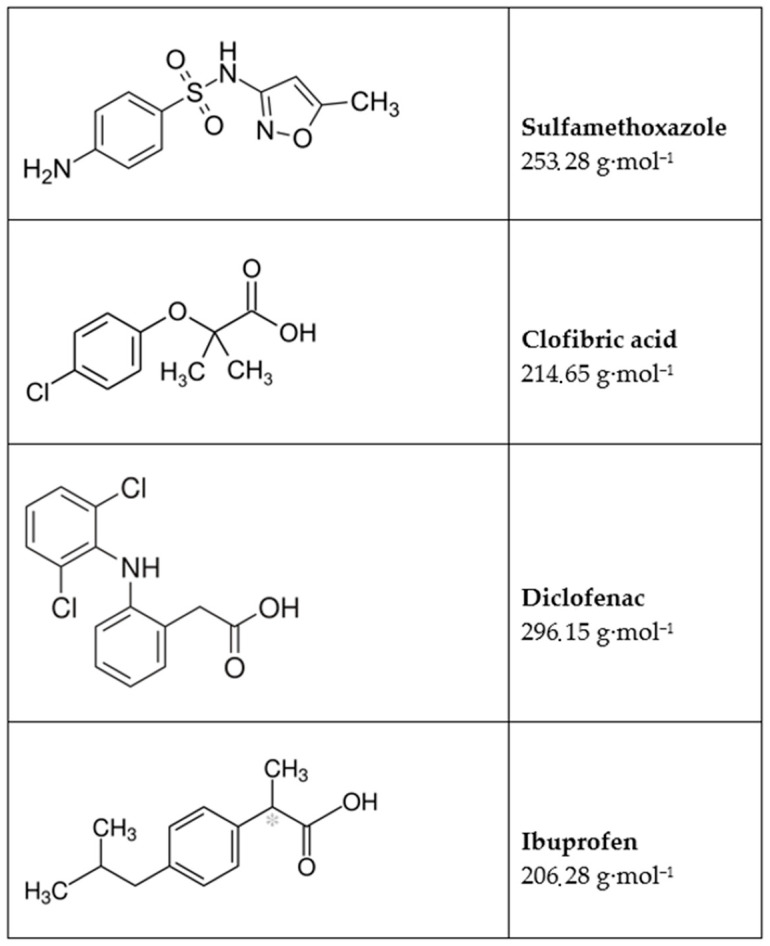
Structure and molecular weight of the tested micropollutants.

The next filtration experiments were performed at 2 bar and 4 bar. The feed solution contained ibuprofen (M = 206 g/mol), clofibric acid (M = 215 g/mol), sulfamethoxazole (M = 253 g/mol) and diclofenac (M = 296 g/mol), each with a concentration of 0.1 mg/L dissolved in ultrapure water. In Figure 12, the molecular structure and molecular weight for the four substances is shown. The biggest molecule is diclofenac, and with exception of sulfamethoxazole all molecules have a negative charge. Sulfamethoxazole has an amine group and is therefore positively charged.

As mentioned in Section 3.1, the ionic strength of the test solutions has an enormous influence on the polyelectrolyte coat. It influences the film-forming properties of the polyelectrolyte as well as the conformation which the polymer has in solution. The concentration polarization due to the applied pressure is counterproductive for the filtration because the formed pores enlarge in this way and do not promote the rejection of small molecules. In the first trials, the micropollutants were dissolved in deionized water which resulted in poor rejection. In order to prevent concentration polarization, the ionic strength of the solution was adjusted using MgSO_4._ The increased number of ions in the solution should lead to stronger interactions with the charged polyelectrolytes. These will knead with each other and therefore form a dense and thicker structure of the surface of the membrane. The ionic strength was adjusted by adding 600 mg/L MgSO_4_ (0.005 M) to the feed solution. For comparison, the system was filtrated first using the simple ceramic membrane without an LbL coat. The received permeate was analyzed using HPLC.

The unmodified ceramic membrane with the smallest pore size of 60 nm was not able to retain the small molecules of the four pharmaceuticals. The rejection rates varied between 3% and 4%.

**Figure 13:**
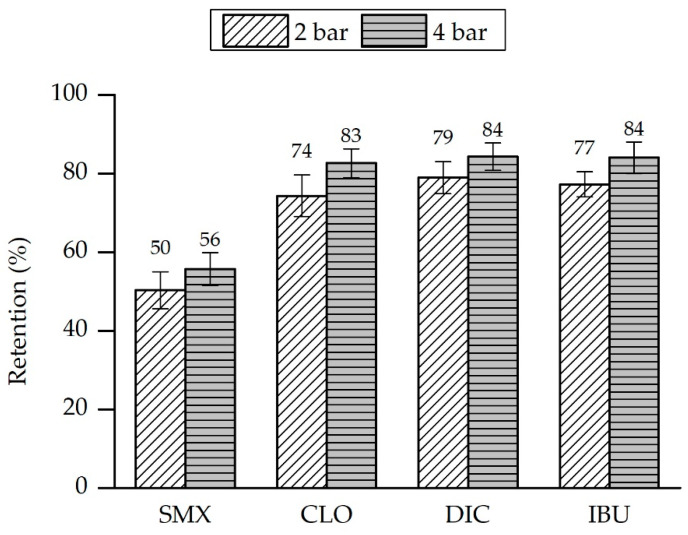
Rejection of pharmaceuticals by a hybrid membrane in feed water with 0.1 mg/L of each micropollutant.

Filtration experiments with the hybrid membrane were the next step in the investigation. The same feed solution was streamed through the LbL-coated membrane and the permeate was characterized. Figure 13 gives an overview of the received results for two different pressure levels. Beginning with a pressure of 2 bar, the rejection rates for the different contaminants are between 50% (sulfamethoxazole) and 79% (diclofenac). The best results were achieved with diclofenac, which has the largest molecules of all tested substances [35]. We observe a slight increase in retention when increasing the pressure to 4 bar. The reason for this might be a slight compaction of the coating which results in the narrowing of the pores. The increased pressure promotes even higher rejection rates for all substances, going up from 56% to 84%. Possible explanations could be compaction of the layers or the larger molecules might form a filter cake on the surface at increased pressure, which leads to the improvement of the rejection.

The lowest rejection is observed for sulfamethoxazole. The obvious explanation here is the positive charge of the molecule in comparison to the negative charge of the other molecules. In this case, the charge of the membrane must be defined by the last layer of polyelectrolyte, which is in this case PSS and the charge is therefore negative.

## 4. Conclusions and Outlook

The experimental development of hybrid membranes began with a selection of ceramic supports for membrane and polyelectrolyte layers. Al_2_O_3_ was selected as support material. The geometries for the tests were mono-channel ceramic membranes (monotubes), which, because of their simplicity, enable easy manufacturing and characterization.

The choice of polyelectrolytes falls on substances that are typically used for LbL coating and delivered good results in previous studies. The fluorescence microscope and STEM analysis confirmed that the polyelectrolytes were able to adhere to the surface of the ceramic membrane and form LbL layers. The measured pore size of the ceramic membrane was between 59 and 215 nm. In total, 10% to 20% of the mass transport in the hybrid membrane occurs through pores with Kelvin radii of less than 1.5 nm, and approx. 84% or approx. 73% of the mass transport takes place in pores with Kelvin radii of greater than 4.4 nm. Molecular weight cut-off measurement was not possible for the uncoated ceramic membranes due to precipitation of the dextran mixture. The molecular weight cut-off for the hybrid membranes was found to be 270 g/mol (≈Da), which corresponds to the MWCO of a nanofiltration membrane. A Zeta potential pH scan confirmed that the application of polyelectrolyte on the surface of the ceramic membranes leads to a switching of the Zeta potential trend and its isoelectric point. The permeability of the hybrid membrane is significantly lower than those of the ceramic membranes and its values are not sensitive to pressure changes.

The performed filtration experiments with feed solution containing ibuprofen, clofibric acid, sulfamethoxazole and diclofenac showed that the ceramic membrane has very low rejection rates for those contaminants (rejection rates between 3% and 4%). The hybrid membrane was able to reject the four contaminants up to 84%. The increase in the pressure improves the rejection of the four pharmaceuticals. Only sulfamethoxazole rejection rates are significantly lower. The reason for this may be its molecule structure and electrostatic interaction with the polyelectrolytes. Sulfamethoxazole bears an amine group and is positively charged at neutral pH. The lower retention implicates that the rejection mechanism is mainly driven by electrostatic repulsion.

This work shows that the LbL coating of ceramic membranes with polyelectrolytes offers a meaningful application for the removal of micropollutants from water. Further investigation envisages experiments with different pharmaceuticals, clarifying the reason for the low rejection rates of sulfamethoxazole and the investigation of the influence of pH and ionic composition of the feed on the filtration process. This also includes the filtration of raw water from different sources. The LbL coating parameters are an important factor for the membrane performance and their influence must be further investigated. A long-term study of the membrane stability should be conducted to clarify the process condition under which the hybrid membrane can be applied in the field of wastewater treatment.

## Figures and Tables

**Table 1:** Pore size characteristics for ceramic membranes.

Maxuimum Pore Size in µm	Mean Flow Pore Size in µm	Minimum Pore Size in µm	Bubble Point Pressure in bar	Bubble Point Flow Rate in L/m
0.2148	0.1275	0.0594	2.9800	0.0678

## Data Availability

The data presented in this study are available on request from the corresponding author. The data are not publicly available due to privacy restrictions.

## Symbols Used

U_str_	mV	Streaming potential
ε_0_	N/A^2^	Vacuum Permeability
ε	S/m	Fluid dielectric constant
η	Pa*s	Viscosity of the fluid
L	m	Length of the rectangular slot channel between two flat surfaces
A	m^2^	Cross section of the rectangular slot channel between two flat surfaces
R	Ω	Electrical resistance in the flow channel
L	L/m^2^·h·bar	Membrane permeability
ϵ_p_	%	Membrane porosity
d_p_	m	Pore size
x	m	Membrane thickness
R	%	Rejection
C_p_	%	Concentration of contaminants in the permeate
C_f_	%	Concentration of contaminant in the feed
A	S/m	Conductivity
L_cond_	L/m^2^·h·bar	Permeability as a function of the conductivity
V	m^3^	Volume
t	s	Filtration time
p	bar	Pressure
Perm_t_	L/m^2^·h·bar	Permeability at filtration time t
Perm_i_	L/m^2^·h·bar	Initial permeability

## Abbreviations

STEM	Scanning Transmission Electron Microscope
DOC	Dissolved Organic Carbon
RO	Reversed Osmosis
LbL	Layer by Layer
PAH	Poly Allylamine Hydrochloride
PEI	Poly Ethyleneimine
PDADMAC	Poly Diallyldimethylammonium Chloride
PVS	Poly Vinyl Sulfate
PAA	Poly Acrylic Acid
PSS	Poly Styrene Sulfonate
TRIS	Tris(hydroxymethyl)aminomethane
GPC	Gel Permeation Chromatography
MWCO	Molecular Weight Cut-Off
PEG	Polyethylen glycol
IBU	Ibuprofen
CLO	Clofibric acid
SMX	Sulfamethoxazole
DIC	Diclofenac
FIB	Focued Ion Beam
HPLC	High-Performance Liquid Chromatography
PMT	Photomultiplier Tube
HAADF	High Angle Annular Dark Field
